# Exosomal circRNAs: key modulators in breast cancer progression

**DOI:** 10.1038/s41420-025-02494-w

**Published:** 2025-04-24

**Authors:** Guozhen Liu, Quan Liu, Lingmei Jia, Zhi Chai, Li Jing, Fangjing Xu, Yucheng Fan

**Affiliations:** 1 Department of Spinal Surgery, General Hospital of Ningxia Medical University, Yinchuan, China; 2 Department of Thyroid and Breast Surgery, The First People’s Hospital of Xiantao, Affiliated Hospital of Hubei University of Science and Technology, Xiantao, China; 3 Department of Anesthesiology and Perioperative Medicine, General Hospital of Ningxia Medical University, Yinchuan, China; 4 Clinical Laboratory Center, Xi’an People’s Hospital Xi’an Fourth Hospital, Affiliated People’s Hospital of Northwest University, Xi’an, China; 5 School of Basic Medical Sciences, Ningxia Key Laboratory of Vascular Injury and Repair, Ningxia Medical University, Yinchuan, Ningxia China; 6 Department of Critical Care Medicine, Yinchuan Hospital of Traditional Chinese Medicine, Affiliated to Ningxia Medical University, Yinchuan City, Ningxia Hui Autonomous Region China; 7 Department of Pathology, The First People’s Hospital of Shizuishan, Affiliated to Ningxia Medical University, Shizuishan City, Ningxia Hui Autonomous Region China

**Keywords:** Cancer genomics, RNA

## Abstract

Breast cancer (BC) poses significant challenges globally, necessitating a deeper understanding of its complexities. Exosomes are cell-specific secreted extracellular vesicles of interest, characterized by a lipid bilayer structure. Exosomes can carry a variety of bioactive components, including nucleic acids, lipids, amino acids, and small molecules, to mediate intercellular signaling. CircRNAs are a novel class of single-stranded RNA molecules, characterized by a closed-loop structure. CircRNAs mainly exert ceRNA functions to intricately modulate gene expression and signaling pathways in breast cancer, influencing tumor progression and therapeutic responses. The unique packaging of circRNAs within exosomes serves as novel genetic information transmitters, facilitating communication between BC cells and microenvironmental cells, thereby regulating critical aspects of BC progression, immune evasion, and drug resistance. Besides, exosomal circRNAs possess the capabilities of serving as diagnostic and therapeutic biomarkers of BC, due to their stability, specificity, and regulatory roles in tumorigenesis and metastasis. Therefore, this review aims to elucidate the novel roles and mechanisms of exosomal circRNAs in BC progression, as well as their potential for diagnosis and therapeutics. The ongoing investigations of exosomal circRNAs will potentially revolutionize treatment paradigms and improve patient outcomes of BC.

## Facts

1. Exosomal circRNAs are crucial modulators in breast cancer progression, influencing tumor growth, metastasis, and drug resistance.
2. The unique packaging of circRNAs within exosomes enables stable intercellular communication for impacting the tumor microenvironment.
3. Exosomal circRNAs have potential as non-invasive diagnostic biomarkers due to their stability and specificity in biofluids.
4. CircRNAs within exosomes can regulate immune responses by modulating macrophage polarization, contributing to immune evasion.

## Open questions

1. How do exosomal circRNAs from non-tumor cells influence breast cancer progression and treatment resistance?
2. What are the specific mechanisms by which exosomal circRNAs modulate immune cell behaviors beyond macrophages?
3. How can we improve the specificity and sensitivity of exosomal circRNAs as diagnostic biomarkers for different breast cancer subtypes?
4. What are the potential therapeutic strategies to target exosomal circRNAs to overcome drug resistance in breast cancer?

## Introduction

Breast cancer (BC) stands as a prevalent and formidable health concern globally, posing significant risks to individual well-being and longevity [1]. Despite advancements in early detection and effective approaches like mastectomy, chemoradiotherapy, and immunotherapy, BC continues to face challenges such as high rates of invasion, metastasis, recurrence, and drug resistance [2, 3]. These obstacles necessitate a deeper understanding of the intricate nature of BC.

Non-coding RNAs (ncRNAs) are a diverse class of RNA molecules found throughout the eukaryotic genome, mainly containing miRNAs, lncRNAs, and circRNAs [4]. NcRNAs have been implicated in pivotal roles in BC development. Among them, circular RNA (circRNA) is a novel class of single-stranded RNA molecules with a covalently closed circular structure, lacking a 5′ cap and a 3′ poly(A) tail [5]. CircRNAs are mainly derived from gene exons or introns, and are widely present in eukaryotes [6]. CircRNAs can serve as miRNA sponges, translation templates, and regulators of gene expression, particularly playing pivotal roles in processes related to various pathogenesis [7]. Most circRNAs are ncRNAs, exhibiting high conservation and overall lower abundance compared to mRNA. CircRNAs are typically tissue-specific and developmentally regulated, exerting significant influence in the progression of BC [8]. For example, circADAM9 showed an overexpressed trend in BC cells, and circADAM9 knockdown suppressed tumor growth in vivo, possibly through the miR-1236-3p/FGF7 axis [9]. Besides, as the resistance to degradation by RNA exonucleases and RNase R, circRNA maintains relative stability in biological samples, offering a dependable and enduring foundation as a biomarker potential [10]. Therefore, the unique structure and multifaceted functions make circRNA a focal point of research, providing new insights into cellular regulation mechanisms and BC pathogenesis.

**Fig. 1:**
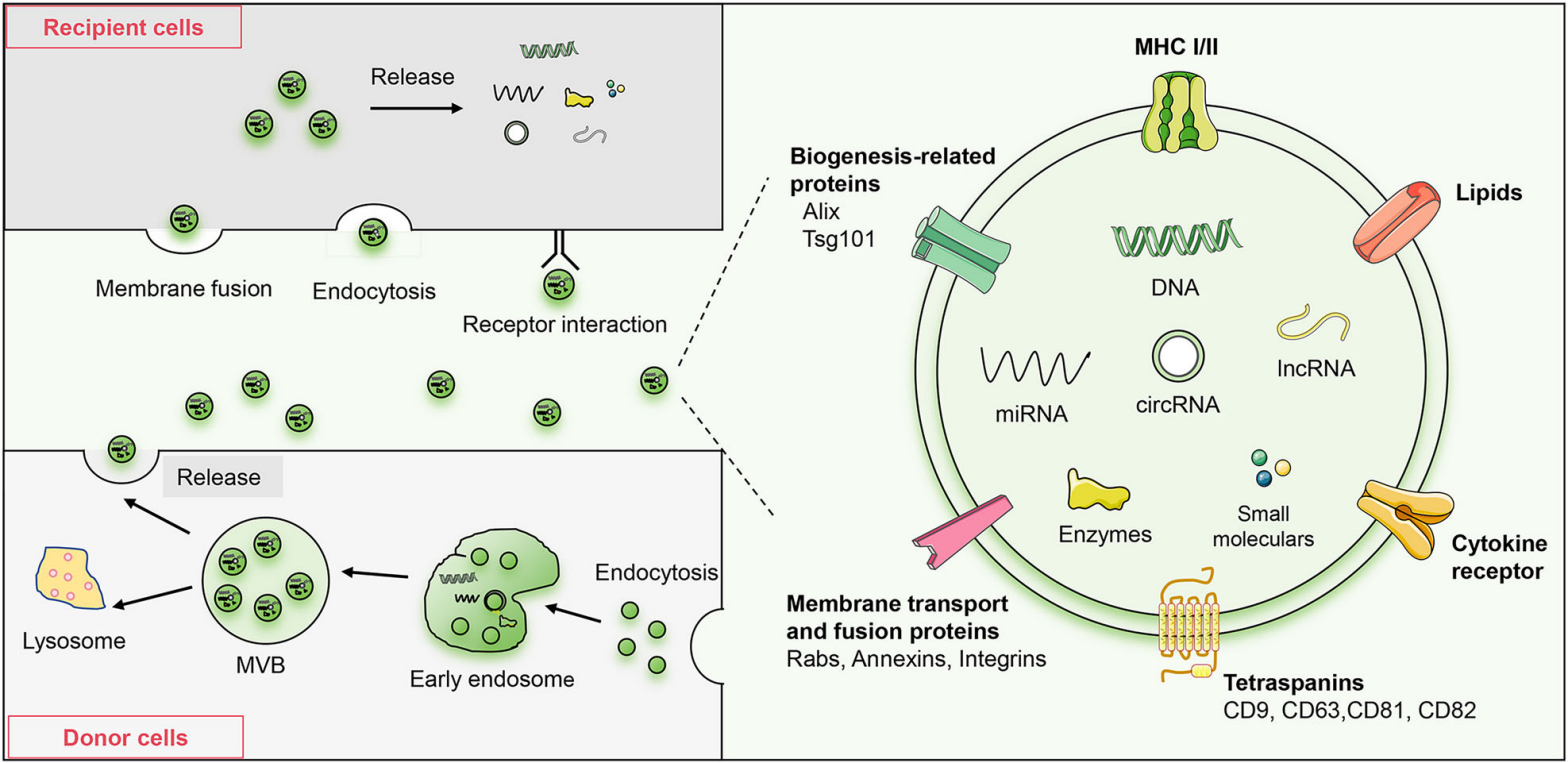
Exosome biogenesis and uptake mechanisms. Exosomes originate from the inward budding of the endosomal membrane, forming early endosomes. These early endosomes mature into multivesicular bodies (MVBs), which then fuse with the plasma membrane to release exosomes into the extracellular space. Exosomes are characterized by a series of conserved proteins and specific biogenesis-related surface biomarkers, such as tetraspanins (CD9, CD63, CD81, CD82), biogenesis-related proteins (Alix, Tsg101), membrane transport and fusion proteins (Rabs, Annexins, Integrins), major histocompatibility complex (MHC), cytokine receptor, lipids. These markers are integral membrane proteins that play crucial roles in exosome stability, function, separation, and identification. Exosomes can be taken up by recipient cells through endocytosis (via clathrin-mediated or caveolin-mediated pathways), direct fusion with membrane, or receptor-ligand interactions involving surface proteins. The cargo of exosomes includes DNA, mRNA messenger RNA, ncRNAs non-coding RNAs, lipids, enzymes, and small molecules, enabling them to modulate a wide range of cellular functions and signaling pathways.

Exosomes, small lipid bilayer-enclosed extracellular vesicles (EVs) ranging from 30 to 150 nm in diameter, contain a diverse array of bioactive molecules, including proteins, nucleic acids, and lipids [11]. Exosomes serve as crucial mediators of intercellular communication, regulating cellular functions and metabolic processes by transferring these bioactive molecules [12]. In tumor microenvironment (TME), exosomes are crucial in promoting tumor cell proliferation, metastasis, and drug resistance, while also influencing immune regulation and shaping the TME [13, 14]. Specifically, exosomes act as key facilitators for the transfer of bioactive molecules, such as circRNAs, between cells in the TME. The packaging of circRNAs within exosomes enables their transmission to neighboring cells, influencing key cellular processes and contributing to disease progression [15]. BC-derived exosomal circRNAs exhibit diverse roles, functioning as both oncogenes and tumor suppressors at various stages of BC progression [16]. CircRNAs within BC-derived exosomes function at different stages of BC progression, modulating critical aspects like immune evasion and drug resistance (Fig. 1).

Therefore, we here conduct an exhaustive literature survey and then decipher the intricate interplay between exosomal circRNAs and BC, investigating their pivotal roles in modulating tumor behavior, shaping immune responses, and influencing drug resistance mechanisms, possibly through exosomal circRNA-miRNA-gene axis regulation. By elucidating the specific contributions of exosomal circRNAs to BC progression, we aim to unveil novel pathways for diagnosing, prognosticating, and treating BC.

## Exosomal circRNAs in regulating BC progression

The TME consists of various stromal cells, including cancer-associated fibroblasts (CAFs), inflammatory cells (such as macrophages, lymphocytes, and granulocytes), endothelial cells, adipocytes, and mesenchymal cells [17]. These cells are crucial in the growth, invasion, and metastasis of tumors, by releasing EVs like exosomes containing bioactive molecules, such as circRNAs, facilitating interactions with tumor cells [18].

### Exosomal circRNAs from tumor cells

Currently, the majority of relevant reports primarily focus on tumor-derived exosomal circRNAs in BC. Through interactions with neighboring tumor cells or stromal cells, these circRNAs affect BC progression by modulating key cellular processes through circRNA-miRNA-target gene regulatory axes [19]. The represented exosomal circRNAs from tumor cells include circSKA3, circPOKE, circRHOT1, circPSMA1, circ_0076611, circHSDL2, circSIPA1L3, and circEGFR, involved in various mechanisms to enhance the malignant behavior and metastasis of BC by acting as competitive endogenous RNAs (ceRNAs), regulating glucose metabolism, and promoting autophagy.

Firstly, the transfer of circSKA3 between BC cells via exosomes enhanced tumor development and invasion, with elevated levels in ascites and heterogeneous expression correlating with increased colony-forming potential, suggesting a predominant role in cell activity [20]. The release of circSKA3-containing exosomes to recipient cells with lower circSKA3 levels regulated colony activities and enhanced the tumor-forming capacity, suggesting a mechanism for maintaining invasive sub-clones in BC. Luo et al. elucidated the role of downregulated circPOKE in BC metastasis by competitively binding to USP10, thus destabilizing Snail and hindering the metastatic potential of BC cells without affecting proliferation [21]. Notably, exosome-mediated delivery of circPOKE significantly impeded BC cell invasiveness in vitro and in vivo. CircRHOT1 showed significant upregulation in serum-derived exosomes from individuals with BC as well as in BC cell lines [22]. Exosomal circRHOT1 derived from BC cells stimulated malignant behaviors such as proliferation, migration, invasion, and epithelial-mesenchymal transition (EMT). Exosomal circRHOT1 was believed to contribute to BC progression by modulating the miR-204-5p/PRMT5 axis. Intriguingly, exosomes from BC cells containing circRHOT1 knockdown exhibited therapeutic potential, attenuating tumor growth in xenograft mouse models. CircPSMA1 exhibited significant upregulation in MDA-MB-231 cells, their exosomes, and triple-negative breast cancer (TNBC) patients, as validated by sequencing [23]. CircPSMA1 acted as a miRNA sponge sequestering miR-637, which directly targeted Akt1 to inhibit migration and metastasis of TNBC cells, impacting downstream genes β-catenin and cyclin D1. TNBC-derived exosomes rich in circPSMA1 could transfer migratory and proliferative capabilities to recipient cells.

Additionally, MALAT1-dependent hsa_circ_0076611 was identified as a regulator of the translation rate in TNBC [24]. Circ_0076611, detected in TNBC cells, tissues, exosomes, and BC patient serum, interacted with proliferation-related transcripts like MYC and VEGFA mRNAs, enhancing TNBC cell proliferation and migration. Mechanistically, circ_0076611 promoted the expression of target mRNAs by facilitating their interaction with translation initiation machinery components, and MALAT1 and ID4 promoted circ_0076611 expression by antagonizing RNA-binding protein PTBP1. Wang et al. extensively examined that circHSDL2 overexpression notably enhanced the activities of BC cells, both in cell culture models and xenografts [25]. Mechanistically, circHSDL2 acted as a ceRNA for miR-7978/ZNF704 axis, thereby modulating the Hippo pathway in BC cells, and enhancing BC aggressiveness.

The interaction of circRNAs with other molecules (such as proteins, DNA, and RNA) or their regulation of target genes can influence glucose, lipid, and protein metabolism [26]. The reprogramming of glucose metabolism is a prominent feature in BC that provides nutrients and energy to support rapid growth and metastasis of tumors, aiding BC cells in adapting to harsh environments [27, 28]. Exosomal circRNAs participate in regulating cellular metabolism and contribute to the pathogenesis of BC. CircSIPA1L3 was identified as a crucial modulator of BC glucose metabolism, and enhanced glycolysis and lactate secretion, thereby fueling tumor progression and promoting the recruitment of tumor-associated macrophages (TAMs) [29]. Mechanistically, EIF4A3 facilitated the cytoplasmic export of circSIPA1L3, stabilizing key glycolytic genes and promoting tumor aggressiveness. Furthermore, circSIPA1L3 stabilized IGF2BP3 and regulated SLC16A1 and RAB11A mRNA expression as a ceRNA. Moreover, circSIPA1L3 was found to be transported by exosomes, contributing to the malignant behavior of BC cells. Elevated circSIPA1L3 expression correlated with poor prognosis and displayed diagnostic potential in BC patients. Liu et al. showed that exosomal circCARM1 derived from breast cancer stem cell (BCSC) spheroids could increase and reprogram cell metabolism by modulating PFKFB2 expression in BC, specifically impacting glycolysis through miR-1252-5p/PFKFB2 pathway [30]. PFKFB2 attenuated the influence of exosomal circCARM1 on cancer cell glycolysis. This investigation highlighted the pivotal role of BCSC exosome-circulated circCARM1 in cellular communication orchestrating metabolic alteration within BC cells. RNA sequencing identified autophagy-responsive circRNA candidates in amino acid-starved TNBC cells, revealing significant upregulation of circEGFR in autophagic cells [31]. CircEGFR knockdown impeded autophagy in TNBC cells, while exosomal circEGFR induced autophagy within TME. Mechanistically, circEGFR promoted ANXA2 translocation, releasing TFEB and promoting autophagy in TNBC cells by disrupting the miR-224-5p/ATG13/ULK1 axis. Besides, plasma exosomal circEGFR exhibited elevated levels in BC patients relative to healthy individuals, together emphasizing the oncogenic role of exosomal circEGFR in TNBC progression and metastasis.

### Exosomal circRNAs from cancer-associated fibroblasts

CAFs are the most abundant and crucial stromal cells in the TME, capable of secreting growth factors, inflammatory ligands, and fibrous proteins, influencing cancer cell activity, therapy resistance, and immune exclusion [32]. CAFs impact tumor progression through paracrine and juxtacrine signaling, ECM-mediated physical cues, and metabolic exchanges, demonstrating their potential value as prognostic factors and therapeutic targets [33, 34]. By releasing exosomes, CAFs not only interact with malignant BC cells but also engage with stromal cells to synergistically regulate the nutritional requirements in various tumor processes [34, 35].

Exosomes from CAFs are crucial in promoting BC progression, partially dependent on fibroblast heterogeneity and the complex composition of exosomes [36]. High-throughput sequencing helps decipher these key factors [37]. High-throughput sequencing revealed the upregulated circTBPL1 expression in exosomes derived from CAFs, compared to normal fibroblasts (NFs) [38]. Transfer of CAF-exosomal circTBPL1 to BC cells promoted cell activities, with circTBPL1 knockdown in CAFs attenuating their tumor-promoting ability. Ultimately, exosomal circTBPL1 derived from CAFs could propagate malignancy and accelerate BC progression via the miR-653-5p/TPBG pathway. CircHIF1A was upregulated in TNBC tissues and promoted tumor progression by enhancing NFIB expression and its nuclear translocation [39]. FUS facilitated circHIF1A biogenesis and was activated by NFIB, forming a positive feedback loop that drove TNBC metastasis. Additionally, circHIF1A was secreted in BC cell-derived exosomes, and was elevated in the plasma of BC patients [39]. Exosomes derived from hypoxic CAFs upregulated circHIF1A, which was transferred to BC cells, enhancing cancer stem cell properties by sponging miR-580-5p and regulating CD44 expression [40]. This study highlighted the pivotal roles of circHIF1A from hypoxic CAF exosomes in modulating BC stemness, suggesting its potential as a therapeutic target for BC. Therefore, circTBPL1 and circHIF1A in exosomes released by CAFs play crucial roles in promoting BC growth and metastasis.

### Exosomal circRNAs from adipocytes

In the TME of BC, cancer-associated adipocytes (CAAs), as adipocytes influenced by the tumor, engage in crosstalk with BC cells, secreting cytokines, lipid molecules, and chemokines to induce alterations in the phenotype and functionality of BC cells [41, 42]. The glucose, lipid, glutathione, and amino acid metabolism of CAAs is also regulated by the surrounding infiltrating BC cells. Moreover, CAAs can modulate malignant alteration of BC through transferring effector molecules via exosomes. Exosomal circCRIM1 was significantly upregulated in TNBC patients with elevated body fat, and could facilitate tumor progression by inhibiting miR-503-5p and activating glycosylation hydrolase OGA [43]. This activation resulted in reduced protein stability of FBP1, which positively correlated with the presence of certain immune cell types within TME. The influence of adipose-derived exosomal circCRIM1 on OGA/FBP1 signaling axis indicates that targeting circCRIM1 in exosomes may provide a novel approach to impede obese TNBC advancement.

## Exosomal circRNAs in BC immune regulation

Exosomal circRNAs, derived from tumor cells or stroma cells, facilitate intercellular communication and significantly impact immune modulation [44, 45]. Among the various immune cells, macrophages are predominantly reported and influenced by these exosomal circRNAs [46]. Upon uptake, these circRNAs can induce macrophage polarization towards a tumor-supportive M2 phenotype, thus enhancing immunosuppressive environments and promoting tumor progression [47]. This mechanism enlightens the feasibility of targeting exosomal circRNAs as a therapeutic strategy to modulate immune responses in BC.

Notably, circ-0100519 was identified as an upregulated biomarker in BC tumor tissues and was related to macrophage infiltration [48]. Tumor-secreted circ-0100519 was transferred to macrophages via exosomes, promoting BC progression by triggering M2 macrophage polarization, for promoting BC cell invasion and metastasis. It concluded that exosomal circ-0100519 drove BC progression by inducing M2 macrophage polarization through the USP7/NRF2 axis. Yang et al. indicated that hsa_circ_002144 was markedly overexpressed in BC tissues, facilitating the proliferation, migration, and EMT of cancer cells while suppressing apoptosis [49]. Moreover, hsa_circ_002144 was found to induce M2 polarization in TAMs via exosomal delivery, influencing glucose metabolism and macrophage polarization. Importantly, hsa_circ_002144 upregulated PKM expression by sequestering miR-326, promoting glycolysis, and enhancing the aggressiveness of BC cells. Lu et al. identified that circ_0001142 was significantly overexpressed in BC tissues and cell lines, with its release from BC cells being enhanced by endoplasmic reticulum stress [50]. They further demonstrated that exosomal circ_0001142 entered macrophages, promoting their M2 polarization through the circ_0001142/miR-361-3p/PIK3CB signaling pathway. Therefore, exosomal circ_0001142 was a crucial orchestrator in modulating TME and facilitating BC progression.

Exosomal circSERPINE2 was overexpressed in BC and facilitated tumor progression by transferring to TAMs, accompanied by high IL-6 production [51]. This interaction triggered a positive feedback loop, where IL-6 promoted EIF4A3 and CCL2 expression in MCF-7 cells by activating the JAK2-STAT3 pathway, and amplifying circSERPINE2 production and TAM recruitment. Targeting circSERPINE2 with siRNA-loaded PLGA nanoparticles significantly inhibited tumor growth in vivo, demonstrating a potential therapeutic approach for BC. In summary, exosomal circRNAs, such as circ-0100519, hsa_circ_002144, circ_0001142, and circSERPINE2 could reshape BC progression by modulating TAMs, promoting M2 polarization, enhancing angiogenesis, through various signaling pathways. Exploring the effects and potential of targeting exosomal circRNAs on macrophages is valuable, but it is also worthwhile to investigate the impact on other immune cells in the TME (Table 1).

**Table 1:** Specific exosomal circRNAs in breast cancer progression.

Exosomal substances	Expression	Donor cells	Recipient cells	Targeted genes/proteins	Function and mechanism	Refs.
circSKA3	high levels in BC patients	BC cells	BC cells	SKA3	increase colony-forming, potentiate tumor invasion, and metastasis	[20]
circPOKE	downregulated in primary and metastatic BC tissues	BC cells	BC cells	USP10-Snail axis	inhibited the invasive capabilities of BC cells in vitro and in vivo	[21]
circRHOT1	high expression in serum of BC patients and BC cells	BC cells	BC cells	miR-204-5p/PRMT5 axis	promote the tumor growth of BC in vivo	[22]
circPSMA1	upregulated in TNBC cells and their exosomes and in TNBC patients	BC cells	BC cells	miR-637/Akt1/β-catenin (cyclin D1) axis	facilitate the tumorigenesis, metastasis, and migration in TNBC	[23]
circ_0076611	detectable in TNBC cells and serum of BC patients	BC cells	BC cells	interact with various proliferation-related transcripts MYC and VEGFA mRNAs	affect the expression of proangiogenic cytokines, control VEGFA expression, and increase TNBC cell proliferation and migration	[24]
circHSDL2	upregulated in BC tissues	BC cells	BC cells	miR-7978/ZNF704 axis and Hippo pathway	contribute to proliferation, migration, and invasion	[25]
circSIPA1L3	upregulated in BC tissues	BC cells	BC cells	coordinate the expression of SLC16A1/RAB11A by sponging miR-665	stabilized IGF2BP3 through facilitating USP7-mediated deubiquitination, thereby contributing to stabilizing SLC16A1 and RAB11A mRNA, and promote progression and glycolysis of BC	[29]
circCARM1	high expression in	BCSC	BC cells	miR-1252-5p/PFKFB2 axis	regulate PFKFB2 expression, increase aerobic glycolysis, promote BC growth	[30]
circEGFR	upregulated in autophagic TNBC cells and patient tissues	BC cells	BC cells	miR-224-5p/ATG13/ULK1 feedback loop	facilitate TNBC autophagy via promoting TFEB nuclear trafficking, promote malignant progression, and metastasis of TNBC	[31]
circTBPL1	increased in CAF exosomes	CAFs	BC cells	miR-653-5p/TPBG	promote BC growth and metastasis	[38]
circHIF1A	upregulated in BC plasma	BC cells	BC cells	circHIF1A/NFIB/FUS positive feedback loop	promote TNBC growth and metastasis	[39]
circHIF1A	upregulated in hypoxic CAF exosomes	CAFs	BC cells	miR-580-5p/CD44	modulate BC cell proliferation and stemness, promote tumor progression	[40]
circCRIM1	upregulated in TNBC patients	adipocytes	BC cells	inhibit miR-503-5p and activate the OGA/FBP1 signaling	promote TNBC growth	[43]
circ-0100519	upregulated in BC tissues	BC cells	macrophages	USP7/NRF2 axis	promotes M2 macrophage polarization and BC progression	[48]
hsa_circ_002144	highly expressed in BC tissues	BC cells	macrophages	miR-326/PKM Axis and the glycolytic pathway	promotes M2 macrophage polarization, BC growth, and metastasis	[49]
circ_0001142	highly expressed in BC tissues	BC cells	macrophages	miR-361–3p/PIK3CB pathway	promote M2 polarization, tumor proliferation, and metastasis	[50]
circSERPINE2	overexpressed in BC	BC cells	macrophages	MALT1-NF-κB-IL-6 axis	promoted IL-6 in TAMs via NF-κB pathway to encourage BC progression	[51]

## Exosomal circRNAs in BC diagnosis

Due to their inherent stability, abundance, and widespread distribution, exosomal circRNAs are considered promising diagnostic and prognostic biomarkers for liquid biopsy applications [52, 53]. Besides, exosomes maintain stability in circulation and protect circRNAs from degradation [54]. Samples for exosomal circRNA analysis can be easily obtained from various biofluids such as blood, urine, and saliva [55]. This makes them a convenient and non-invasive approach to biomarker discovery. Circulating circRNAs packaged in exosomes offer a novel avenue for non-invasive diagnosis of BC [56]. The detection of exosomal circRNA in diverse biofluids serves as a rich source of molecular information for elucidating the intricate molecular landscape during BC progression, hence providing crucial insights into BC diagnosis and responses to therapeutic interventions.

RNA-seq unveils novel specific circRNAs implicated in BC progression and patient therapy response [57]. A ceRNA regulatory network was successfully constructed from exosomes of human BC patients, revealing 42 differentially expressed mRNA, 43 circRNA, and 26 lncRNA [58]. The network included 19 mRNA nodes, 2 lncRNA nodes, 8 circRNA nodes, and 41 miRNA nodes, with enrichment analysis highlighting involvement in the p53 signaling pathway and identification of the key Hub gene PTEN, offering potential targets for BC diagnosis and treatment. Zhu et al. successfully constructed a ceRNA regulatory network from exosomes of human BC patients, revealing 42 differentially expressed mRNA, 43 circRNA, and 26 lncRNA [58]. Mao et al. identified 347 upregulated and 3 downregulated circRNAs in BC patients. A diagnostic model based on 14 circRNAs exhibited high diagnostic accuracy [59]. The circRNAs targeted 70 miRNAs and 1147 mRNAs involved in tumor-related pathways like PI3K-AKT, MAPK, RAS, and RAP1. Thus, this exosomal circRNA-based diagnostic model showed promise for BC diagnosis.

Dysregulation of circRNAs is observed in the exosomes derived from BC cell lines, metastatic, and localized BC patients, highlighting the distinct patterns of circRNA expression in different resources of BC samples [60]. For example, Yang et al. revealed significant differential expressions of hsa-circRNA-0005795 and hsa-circRNA-0088088 in serum exosomes and tissues of BC patients, indicating their roles as pivotal diagnostic potential in BC tumorigenesis and metastasis [61]. In Lin et al. study, the differential expression analysis revealed 439 circRNAs in plasma EVs with significantly altered levels between BC patients and controls, showing the potential of exosome circRNA profiles in plasma EVs as valuable biomarkers for early BC detection [62]. Subsequently, the development and validation of BC_ExoC_, a nine-circRNA combined classifier utilizing an SVM model, demonstrated a robust predictive efficacy with an AUC of 0.83 in the training cohort and 0.80 in the validation cohort. These results confirmed the promising role of exosomal circRNAs as non-invasive liquid biopsies for BC diagnosis and management.

The elevated expression of has_circ_0000615 in plasma samples could distinguish BC patients from healthy controls, displaying substantial links to advanced tumor stage, lymph node metastasis, and high recurrence risk grade [63]. The diagnostic value of has_circ_0000615 for non-metastatic BC was notable, with an AUC of 0.904, sensitivity of 76.8%, and specificity of 88.4%, surpassing conventional tumor biomarkers like CA153, CA125, and CEA. Exosome analysis revealed the presence of has_circ_0000615 in exosomes, indicating its potential role as a diagnostic marker secreted into the circulation of BC patients. In a meta-analysis of 17 studies involving 1234 BC patients post-surgery, exosomal circRNAs demonstrated a pooled sensitivity of 0.85 and specificity of 0.83 for detecting wound healing complications, highlighting their high diagnostic accuracy in monitoring post-operative wound healing [61]. These results confirm the potential of exosome-based circRNAs as promising non-invasive biomarkers for guiding personalized patient management in BC recovery.

## Exosomal circRNAs in drug resistance of BC

While treatment modalities for BC, including surgery, endocrine therapy, and targeted therapy, continue to advance, drug resistance remains a formidable challenge [64, 65]. Common mechanisms of BC drug resistance include alterations in drug targets, reshaping of cell signaling pathways, changes in TME, multidrug resistance, and enhanced DNA repair mechanisms [66]. Frequently encountered drug resistances involve endocrine therapy agents (tamoxifen), HER2-targeted therapies (trastuzumab, lapatinib), chemotherapy drugs (paclitaxel (PTX), doxorubicin (DOX)), PARP inhibitors (olaparib), and CDK4/6 inhibitors (palbociclib, abemaciclib) [67, 68]. The presence of these resistance mechanisms complicates BC treatment, necessitating the exploration of novel therapeutic strategies to address drug resistance challenges. Moreover, exosomes within the TME serve as mediators of transmission, facilitating both tumor drug resistance and therapeutic interventions, with their cargoes of circRNAs playing a pivotal genetic role in drug resistance mechanisms.

PTX is a well-known first-line chemotherapy drug that induces tumor cell mitotic arrest and apoptosis, inhibits angiogenesis, and modulates immune responses [69, 70]. However, resistance to PTX treatment remains a significant challenge in BC, with mechanisms involving overexpression of multidrug resistance proteins, mutations in tubulin proteins, and abnormalities in apoptotic pathways [71]. Li et al. showed that the upregulation of circ_0001955 in BC tissues and cell lines, including its presence in exosomes from BC cell lines, impeded proliferation, diminished PTX sensitivity, and disrupted glycolytic processes in MCF-7 and MDA-MB-231 cells [72]. Deletion of circ_0001955 inhibited tumor growth in vivo, directly interacting with miR-708-5p and indirectly modulating PGK1, regulating BC progression. Therefore, the exosomal release of circ_0001955 from BC cells boosted cell proliferation and glycolysis, affecting PTX sensitivity through miR-708-5p-induced PGK1 upregulation. CircBACH1 was upregulated in PTX-treated BC-derived exosomes (PTX-EXO) and tissues, promoting PTX chemoresistance and angiogenesis by sponging miR-217 to enhance the expression of G3BP2 [73]. Silencing circBACH1 reversed these effects, enhancing PTX sensitivity. Thus, targeting PTX-induced exosomal circBACH1 and its axis circBACH1/miR-217/G3BP2 were novel strategies in combating BC and PTX resistance.

DOX, belonging to an anthracycline chemotherapy drug, is widely used in treating BC and other malignancies [74]. Conventional DOX faces challenges due to its non-selectivity, leading to therapeutic failures and adverse effects. Exosomes facilitate the transfer of ncRNAs, such as miR-181b-5p, leading to the development of DOX resistance in BC, highlighting a sophisticated mechanism of intercellular communication in drug resistance pathways [75]. circRNA-CREIT was found to downregulate PKR through E3 ligase HACE1-mediated degradation, inhibiting PKR/eIF2α signaling and stress granule formation in TNBC [76]. Exosomal circRNA-CREIT transmission enhanced DOX sensitivity among TNBC cells. DHX9-mediated regulation of circRNA-CREIT biogenesis was associated with a favorable prognosis in TNBC, suggesting a potential therapeutic strategy against DOX chemoresistance.

Pirarubicin (THP) is a widely utilized anthracycline chemotherapy agent, and exhibits potent anticancer properties effective in treating diverse cancers, notably BC [77]. THP exerts its anticancer effects by inhibiting topoisomerase II, generating free radicals, inducing apoptosis, and disrupting DNA function in cancer cells [78]. In Zhang et al. investigation, the high expression of circZCCHC2 was detected in plasma-originating exosomes, TNBC cells, and TNBC tissues [79]. The heightened expression of circZCCHC2 exhibited a robust correlation with tumor size. The suppression of cirZCCHC2 activity hindered TNBC cell proliferation, migration, invasion, and EMT both in vitro and in vivo. Treatment with THP reduced cirZCCHC2 levels, impacting THP sensitivity, thereby unveiling circZCCHC2 role in TNBC progression. This regulation of circZCCHC2 through the miR-1200/TPR axis, activated the RAS-RAF-MEK-ERK pathway, thus posing a potential axis for intervening TNBC progression.

Tamoxifen, a pivotal endocrine therapy agent in BC treatment, competes with estrogen receptors, halting estrogen tumor-promoting effects [80]. Exosomal-mediated substances, notably miR-22 and miRNA-205, play pivotal roles in the development of tamoxifen resistance in BC cells [81, 82]. Exosomes significantly transferred circ-UBE2D2, which was upregulated in tamoxifen-resistant BC tissues and cell lines, enhancing resistance by interacting with miR-200a-3p [83]. The interaction modulated BC cell viability, migration, and ERα expressions. This study implied that the exosomal shift of circ-UBE2D2 strengthened tamoxifen resistance in BC cells through its interaction with miR-200a-3p, revealing a new approach to enhance tamoxifen efficacy.

Lapatinib is an orally active small-molecule tyrosine kinase inhibitor that targets human EGFR1 and HER2 [84]. Clinical trial results demonstrate that lapatinib is not only active in HER2 + BC but also shows potential in the treatment of metastatic BC and patients with brain metastases. Circ-MMP11 was confirmed to be upregulated in lapatinib-resistant BC tissues and cells, compared to normal MCF-10A [85]. Circ-MMP11 was transferred via exosomes and contributed to drug resistance by sponging miR-153-3p to enhance ANLN expression. The knockdown of circ-MMP11 significantly increased lapatinib sensitivity by reducing cell viability, migration, and invasion, implying targeting circ-MMP11/ miR-153-3p/ANLN axis in lapatinib resistance of BC (Table 2).

**Table 2:** Specific exosomal circRNA in diagnosis and drug treatment of breast cancer.

Exosomal substances	Dysregulation	Targeted gene	Clinical values	Refs.
hsa-circRNA-0005795	low expression in serum exosomes from BC patients	hsa-miR-1304-3p, hsa-miR-3154	BC prognosis and therapy	[61]
hsa-circRNA-0088088	high expression in serum exosomes from BC patients	let-7a-2-3p	BC prognosis and therapy	[61]
has_circ_0000615	high expression in serum exosomes from BC patients	not applicable	BC prognosis	[63]
circ_0001955	highly expressed in BC tissues and cell lines	miR-708-5p/PGK1 axis	facilitate proliferation and glycolysis and enhance the IC50 value of PTX in breast cancer cells	[72]
circBACH1	upregulated in PTX-EXO and tissues	miR-217/G3BP2 axis	Promote stemness and migration of BC cells, a target for PTX-resistance and progression of BC	[73]
circRNA-CREIT	downregulated in doxorubicin-resistant TNBC cells	enhanced HACE1-PKR through ubiquitin-proteasome system	enhance DOX sensitivity of TNBC, a target for poor prognosis	[76]
circZCCHC2	upregulated in TNBC cells, tissues, and plasma exosomes	miR-1200/TPR	decrease pirarubicin sensitivity and promote TNBC development	[79]
circ-UBE2D2	upregulated in tamoxifen-resistant tissues and cell lines	miR-200a-3p	reinforce tamoxifen resistance in BC, modulated BC cell viability, migration, and ERα expressions	[83]
circ-MMP11	highly expressed in LR breast cancer tissues and cells	miR-153-3p/ANLN Axis	significantly increase lapatinib sensitivity by reducing cell viability, migration, and invasion in BC	[85]

## Discussion

The investigation into exosomal circRNAs as pivotal modulators has opened up new avenues for understanding the complexities of BC [86]. This domain is still in its fledgling stage, and there are still some limitations and challenges that need to be addressed in terms of the mechanisms of circRNAs, diagnostic methods, therapeutic strategies, and the clinical application of circRNAs.

Firstly, there are still many gaps in the research on the mechanisms. Currently, research on exosomal circRNAs in BC remains relatively scarce, with only a few dozen articles available. The identification of specific targets of exosomal circRNAs in BC and their impact on cellular functions and behaviors is still lacking. Further analysis of the sequenced results and the use of sequencing technology on different sources of BC samples are needed to discover new exosomal circRNA targets and their functions [87]. Moreover, the regulatory mechanisms governing the generation and release of exosomal circRNAs from various cell types remain inadequately understood, affecting our comprehension of intercellular communication and the dynamic changes within the TME. The specific roles of exosomal circRNAs in metabolic pathways and energy utilization in BC cells are insufficiently explored, hindering the elucidation of metabolic regulation mechanisms [88].

Secondly, existing research primarily focuses on tumor-derived exosomal circRNAs, yet those from other TME components like immune cells, fibroblasts, and adipocytes are increasingly recognized for their crucial roles in BC progression, highlighting the need for further sequencing and experimental validation [89]. In addition to tumor-derived exosomal circRNAs, those originating from other components of TME, such as immune cells, fibroblasts, and adipocytes, are increasingly recognized for their pivotal roles in BC progression. These circRNAs synergistically and indispensably contribute to the intricate network of BC biology by modulating immune responses, facilitating stromal interactions, and reprogramming cellular metabolism. For example, CAFs and adipocytes release exosomal circRNAs like circTBPL1, circHIF1A, and circCRIM1, which significantly impact BC progression by modulating tumor growth, metastasis, and metabolic pathways. Additionally, exosomal circRNAs such as circ-0100519, hsa_circ_002144, and circSERPINE2 play crucial roles in immune regulation by influencing macrophage polarization and promoting an immunosuppressive TME. For immune regulation, while exosomal circRNA mainly influences macrophages and M2 polarization, the regulatory effects of other immune cell populations in the BC microenvironment and their impact on immune responses and BC progression are areas that still need exploration. And, circHIPK3 is highly expressed in BC cell exosomes and promotes angiogenesis and tumor progression in endothelial cells by regulating the miR-124-3p/MTDH axis [90]. However, exosomal circRNAs from other non-tumor cell types with TME, such as endothelial cells, neural cells, and stem cells, remain insufficiently studied [91]. Totally, exosomal circRNAs from non-tumor and tumor sources may profoundly influence each other through intricate signaling cross-talk, reshaping TME, and altering cellular dynamics and response mechanisms. A comprehensive understanding of these non-tumor cell-derived exosomal circRNAs is essential to figure out their multifaceted and complicated roles in BC, thereby devising innovative therapeutic strategies and refining diagnostic methodologies.

In the diagnosis of BC, the application of exosomal circRNA as a potential biomarker still has some deficiencies. Firstly, although exosomal circRNAs present preliminary potential in BC diagnosis, their specificity and sensitivity require further retrospective and prospective clinical validation [92]. Secondly, exosomal circRNAs possess complex and diverse origins, making it challenging to accurately distinguish their sources and determine their association with BC [93]. Additionally, the application of exosomal circRNAs as biomarkers in BC diagnosis also faces issues of standardization and calibration [94]. The lack of uniform standard operating procedures and quality control standards may lead to instability of results and poor reproducibility. Furthermore, the dynamic characteristics of exosomal circRNAs and their expression patterns in different subtypes and pathological stages of BC have not been fully elucidated, limiting their precise application in diagnosis [95]. For instance, single-cell RNA sequencing results reveal the dynamic changes in metabolic markers during early BC metastasis, particularly highlighting the transition between glycolysis and oxidative phosphorylation at the onset of metastasis, emphasizing the significance of these dynamic markers in BC progression [96]. Therefore, future research needs to standardize detection methods and explore biomarker dynamics and subtype associations to improve exosomal circRNA accuracy in BC diagnosis.

In the treatment of BC, exosomal circRNAs may participate in drug resistance mechanisms by regulating drug transport, apoptosis escape, DNA repair, and other pathways in tumor cells [97–99]. However, the exact mechanism of exosomal circRNAs in drug resistance is not yet clear, requiring further molecular biology and cell biology research to elucidate their detailed mechanism. Additionally, in terms of resistance of BC patients to other treatment modalities such as chemotherapy, hormone therapy, and radiotherapy, exosomal circRNAs may affect the efficacy by modulating TME immune suppression, angiogenesis, and other pathways [16, 100]. Therefore, studying the role of exosomal circRNAs in BC resistance to other treatment modalities can help develop new combination treatment strategies and improve treatment outcomes [101]. In terms of targeted therapy, exosomal circRNAs may influence the response of BC cells to targeted drugs by regulating the activation or inhibition of signaling pathways [102, 103]. Therefore, investigating how to utilize exosomal circRNAs as auxiliary biomarkers for targeted therapy is of great clinical significance for the individualized and precise treatment of BC patients.

Lastly, the studies on exosomal circRNAs in BC primarily focus on cell and animal models, with limited large-scale clinical trials conducted. The lack of extensive clinical research data from large samples and multiple centers creates uncertainties regarding the potential application of exosomal circRNAs in clinical practice for BC. In the aspect of diagnostic applications, the diverse and complex origins of exosomal circRNAs may be influenced by intra- and extracellular environmental factors, leading to inconsistent and unstable detection results in clinical samples [104, 105]. Additionally, the current standardization level of detection technologies is not high, resulting in variations between different laboratories and methods, which impacts the reliability of exosomal circRNAs as biomarkers [106, 107]. It is crucial to address the limitations and challenges related to the stability, specificity, standardization of detection technologies, and mechanisms of action of exosomal circRNAs as biomarkers and therapeutic targets to further advance and apply them in clinical practice for BC.

In addition to these challenges, the clinical application of exosomal circRNA in BC faces significant regulatory hurdles. Ensuring the safety, efficacy, and reproducibility of these biomarkers requires rigorous validation procedures mandated by regulatory agencies. For BC diagnostics and therapeutics, standardized protocols for exosome isolation, circRNA quantification, and data interpretation are essential to maintain consistency across laboratories [108]. Technical and ethical issues related to circRNA detection must also be resolved to meet regulatory standards. Standardization remains a critical issue, as differences in isolation techniques, quantification methods, and data normalization can affect the reliability of exosomal circRNAs as BC biomarkers. Establishing unified guidelines for sample collection, processing, and analysis is crucial. International and interdisciplinary collaboration is vital for developing these standardized protocols and quality control measures, promoting technical consistency, and facilitating large-scale studies. Although no specific clinical trials have been conducted yet in BC, future research should focus on rigorous trial design to validate diagnostic accuracy, prognostic significance, and therapeutic potential. Advances in high-throughput sequencing and bioinformatics will further aid in discovering and validating new circRNA biomarkers specific to BC, enhancing diagnostic precision and enabling targeted therapeutic strategies, ultimately improving patient outcomes and quality of life.

## Conclusion

**Fig. 2:**
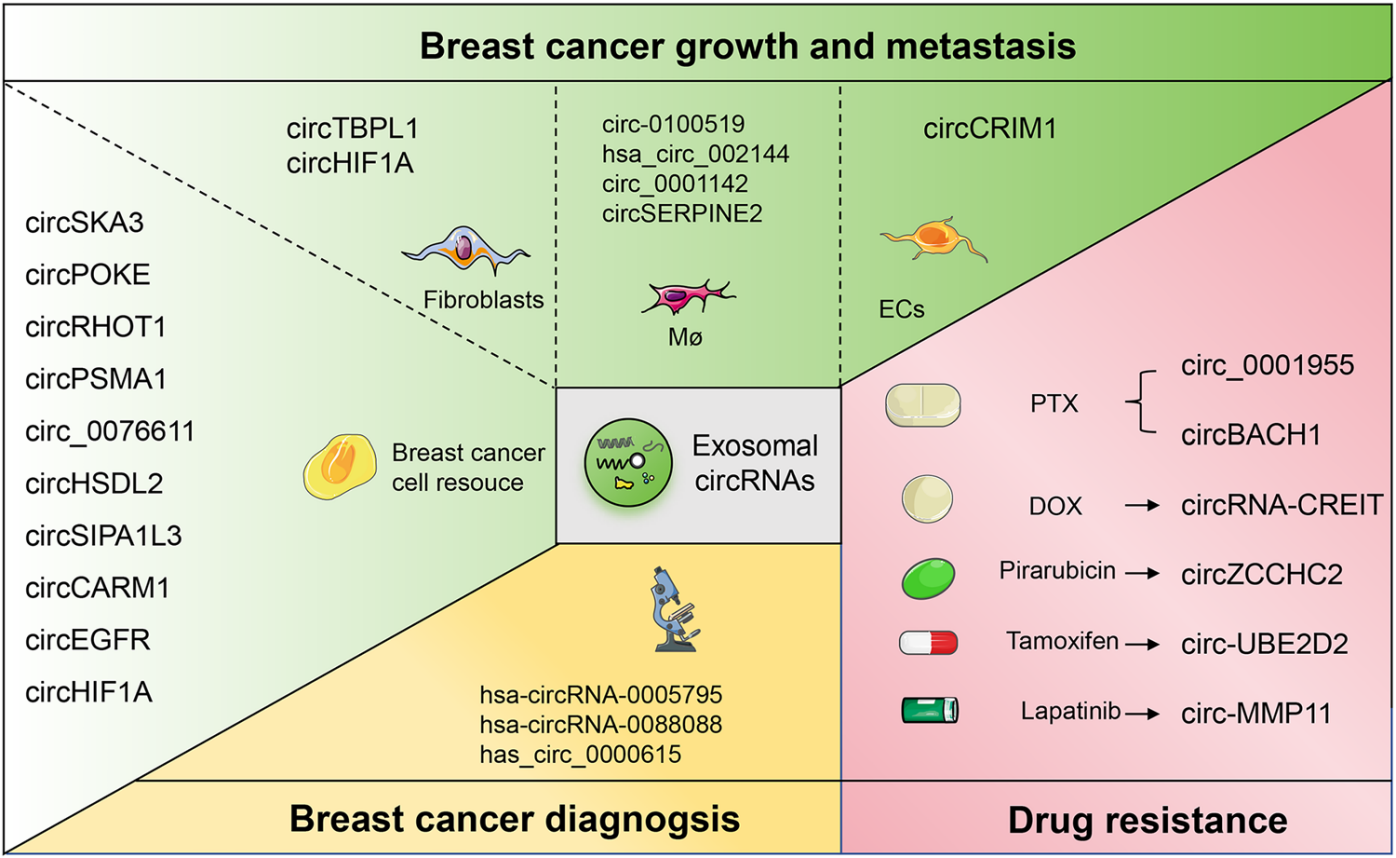
The multifaceted roles of exosomal circRNAs in breast cancer progression, diagnosis, and drug resistance. In cancer progression, circRNAs such as circSKA3, circPOKE, and circRHOT1 enhance tumor invasion, metastasis, and proliferation by modulating pathways like the USP10-Snail axis and miR-204-5p/PRMT5 axis. CircPSMA1 and circHSDL2 contribute to tumorigenesis and migration through interactions with key cellular pathways, including the miR-637/Akt1/β-catenin axis. Additionally, circSIPA1L3 and circCARM1 are involved in metabolic reprogramming, promoting glycolysis and tumor growth. In diagnosis application, circRNAs such as hsa-circRNA-0005795 and has_circ_0000615 emerge as promising biomarkers due to their differential expression in breast cancer patients. For drug resistance, circRNA-CREIT and circBACH1 play pivotal roles in modulating the sensitivity of breast cancer cells to treatments like paclitaxel and doxorubicin, impacting pathways such as the miR-217/G3BP2 axis. These circRNAs are integral to the modulation of tumor behavior, immune regulation, and therapeutic responses, providing valuable insights and targets for innovative breast cancer diagnostics and treatment strategies.

Collectively, we comprehensively investigate and confirm exosomal circRNAs as key modulators in BC progression, by primarily functioning as ceRNAs. Specifically, our findings unveil a novel molecular landscape of exosomal circRNAs that intricately modulate intercellular signaling pathways and gene expressions, consequentially influencing tumor behavior, immune regulation, drug resistance, and diagnostic potential (Fig. 2). However, the mechanisms of exosome circRNAs underlying their biogenesis, release, and intercellular communication remain inadequately understood. Current research predominantly focuses on tumor-derived circRNAs, with insufficient attention given to those originating from immune cells, fibroblasts, and other components within TME, limiting the understanding of their complex roles in the tumor ecosystem. In diagnostic applications, exosomal circRNAs are promising biomarkers due to their stability and specificity, but their specificity and sensitivity require validation through larger clinical studies. Additionally, standardization of detection methods and reproducibility of results are critical challenges that need to be addressed to ensure reliable clinical application. Therapeutically, exosomal circRNAs may contribute to drug resistance mechanisms, while the precise molecular mechanisms remain to be elucidated. By overcoming these challenges, exosomal circRNAs hold the potential to significantly advance precision medicine in BC, revolutionizing both diagnosis and treatment.
